# Draft Genome Sequence of Lactobacillus fermentum Lf2, an Exopolysaccharide-Producing Strain Isolated from Argentine Cheese

**DOI:** 10.1128/MRA.01072-18

**Published:** 2018-11-08

**Authors:** Hugh M. B. Harris, Elisa C. Ale, Jorge A. Reinheimer, Ana G. Binetti, Paul W. O’Toole

**Affiliations:** a School of Microbiology and APC Microbiome Institute, University College Cork, Cork, Ireland; b Instituto de Lactología Industrial (UNL-CONICET), Facultad de Ingeniería Química (UNL), Santa Fe, Argentina; University of Delaware

## Abstract

Lactobacillus fermentum Lf2, an Argentine cheese isolate, can produce high concentrations of exopolysaccharides (EPS). These EPS were shown to improve the texture and rheology of yogurt, as well as to play a protective role in mice exposed to Salmonella enterica serovar Typhimurium. Three gene clusters potentially involved in EPS production were identified in different locations of the L. fermentum Lf2 genome.

## Abstract (precis)

Lactobacillus fermentum Lf2, an Argentine cheese isolate, can produce high concentrations of exopolysaccharides (EPS). These EPS were shown to improve the texture and rheology of yogurt, as well as to play a protective role in mice exposed to Salmonella enterica serovar Typhimurium.

## ANNOUNCEMENT

Lactobacillus fermentum Lf2 was isolated as a nonstarter lactic acid bacterium (NSLAB) from a regional Argentine Tybo cheese that presented blowing defects due to the gas production (CO_2_) of this strain. It was isolated through enumeration of nonspecific gas-producing bacteria in de Man-Rogosa-Sharpe (MRS) broth containing Dürham tubes by the limiting dilution method, which consists of enumerating the highest dilution which presents gas production (1). Its most interesting feature resides in its ability to produce large amounts of exopolysaccharides (EPS) when it is grown under controlled conditions, reaching ∼1 g/liter of crude extract (2).

This EPS extract presented interesting technological properties, providing yogurts with increased consistency and hardness (in concentrations of 300 and 600 mg/liter) without negatively affecting the sensory characteristics of the yogurts (2). Besides, the EPS extract showed a functional role when it was added to milk or yogurt, being able to protect mice against infection from Salmonella enterica serovar Typhimurium and to modulate the immune system at the intestinal level (3).

Total DNA was isolated from 1 ml of an overnight culture, grown from a single colony in MRS broth (37°C, 5% CO_2_), using a DNeasy blood and tissue kit (Qiagen, Hilden, Germany) according to the manufacturer’s instructions. The genome of L. fermentum Lf2 was sequenced by GenProbio (University of Parma, Italy), using 2 × 250-bp paired-end Illumina MiSeq reads. Raw reads (220.77 Mb) were assembled using Velvet (v 1.2.10) using a kmer value of 91 and –exp_cov and –cov_cutoff set to “auto.” Raw reads were untrimmed because this step decreased the quality of the assembly summary statistics when performed using Trimmomatic (v 0.36). The resulting assembly was composed of 251 contigs, with an *N*_50_ of 32,695, average nucleotide coverage of 57×, GC content of 51.7%, and total length of 2.05 Mb. The draft genome was reordered using the Mauve contig mover to match the synteny of the complete genome, L. fermentum CECT5716. Three software packages were used to predict genes, namely, Glimmer3 (v 3.02; parameters, training set and subsequent gene predictions were generated using the program g3-iterated.csh), metagene (v0.1; default parameters), and GeneMark.HMM (v 2.0; parameters, –type heu_11_57.mod –gc 57 from the program gmhmmp_heuristic.pl). A total of 2,804 genes were predicted (2,644 complete and 160 partial), where a gene prediction was kept if at least one software program predicted it; if the same gene (stop codon position) with different lengths (upstream start codon positions) was predicted by either two or three software programs, the longest prediction was kept. Three gene clusters potentially involved in EPS production were identified in different genomic regions. The function of each potential EPS-related gene (those whose predicted functions were associated with EPS biosynthesis) was confirmed by considering the best hit obtained by a BLASTx search against the current NCBI database. Cluster 1 contained 8 genes, including a gene that codifies a capsular polysaccharide synthesis protein, while clusters 2 and 3 contained 15 and 5 genes, respectively.

The chemical characterization of the EPS is being analyzed with the collaboration of Andrew Laws and his group (University of Huddersfield, United Kingdom). From preliminary results, it was possible to elucidate that the EPS of L. fermentum Lf2 is a mixture that is composed mainly of a homopolysaccharide (a β-glucan) and a heteropolysaccharide with glucose and galactose in its structure. This last result was expected, since L. fermentum Lf2 presents two genes (GenBank accession numbers NZ_QLNN01000005 and NZ_QLNN01000066) coding for two priming glycosyltransferases, enzymes that are known to play a key role in the synthesis of heteropolysaccharides.

### Data availability.

This whole-genome shotgun project was deposited at DDBJ/ENA/GenBank under accession number QLNN00000000. The version described in this paper is version QLNN01000000. Raw reads were deposited in the Sequence Read Archive under accession number SRP159489. Both raw reads and the assembled genome are deposited under BioProject number PRJNA476494.
